# Detection of *Helicobacter pylori* infection in children using rapid urease and histologic methods of diagnosis

**DOI:** 10.4314/gmj.v58i1.10

**Published:** 2024-03

**Authors:** Taiba J Afaa, Nana A H Seneadza, Afua D Abrahams, Victor K Etwire, Eric Odei

**Affiliations:** 1 Department of Child Health, University of Ghana Medical School, College of Health Sciences, University of Ghana; 2 Department of Community Health, University of Ghana Medical School, College of Health Sciences, University of Ghana; 3 Department of Pathology, University of Ghana Medical School, College of Health Sciences, University of Ghana; 4 Department of Surgery, Paediatric Surgery Unit, Korle Bu Teaching Hospital; 5 Public Health Department, Korle Bu Teaching Hospital

**Keywords:** Helicobacter pylori, endoscopy, children, rapid urease test

## Abstract

**Objective:**

The study aimed to detect the presence of *Helicobacter pylori* infection in children using two investigative methods: the rapid urease test and histological methods. It also examined the relationship between socioeconomic status and Helicobacter pylori infection.

**Design:**

This was a cross-sectional study conducted in the paediatric theatre at Korle Bu Teaching Hospital in Accra, Ghana.

**Participants:**

Children who were scheduled for upper gastrointestinal endoscopy were recruited into the study.

**Main outcome measures:**

The presence of Helicobacter pylori in gastric biopsies was measured using a rapid urease test and histology.

**Results:**

Seventy-three children aged 2 years to 16 years were seen during the period. Both tests were positive at the same time in 36 (49.3%) out of the 73 children (p<0.0001). The positivity rates for the rapid urease test and histology were 57.5% and 53.4 %, respectively. Significant predictors of the histology presence of H. pylori were a large household size of at least 6 members (AOR: 4.03; p<0.013) and the presence of pets at home (AOR: 3.23; p<0.044).

**Conclusions:**

Substantial agreement was found between the rapid urease test and histology examination of gastric biopsies for the presence of *H. pylori*. Children from large households and those with pets at home appear to have increased odds of having *H. pylori* infection of the gastric mucosa.

**Funding:**

None declared

## Introduction

*Helicobacter pylori (H. pylori)* is one of the most common worldwide human infections.^1^ About 50% of adults the world over are infected with this pathogen.^2^ The vast majority of individuals acquire this infection during childhood.^3^ The infection is largely asymptomatic in children. In symptomatic cases, the clinical features include epigastric pain, persistent vomiting, iron deficiency anaemia, malnutrition, gastrointestinal bleeding and chronic gastritis.^4^ Compared with adults, peptic ulcer disease (PUD) is found less often in infected children undergoing upper gastrointestinal endoscopy.^5^

There are invasive and non-invasive methods of diagnosing the infection.^6^ The invasive endoscopic tests include histology, rapid urease test (RUT), culture, and polymerase chain reaction. Those that do not require endoscopy include serum antibody testing, Urea breath tests and faecal antigen tests. All these tests are suitable for the detection of infection before and after treatment, with the exception of serology, which may remain positive for some time after successful eradication.^7^ For the accurate interpretation of test results, factors that can lead to false-positive or false-negative results must be known and considered. Antibiotics, including penicillin and cephalosporins, and acid-suppressive drugs, particularly proton pump inhibitors (PPIs), should be discontinued for at least 4 and 2 weeks, respectively before testing.^8^ ^9^

This study aimed to detect the presence of *H. pylori* infection using two different invasive methods (RUT and histology) and to study the relationship between indicators of poor socioeconomic status and *H. pylori* infection in these children. The histology test is known to have greater than 95% sensitivity and specificity, while the RUT has 95% and 85% sensitivity and specificity, respectively. The rationale for performing more than one diagnostic test was based on the recommendation of the ESPGHAN/NASPGHAN guidelines for the management of *H. pylori* in children and adolescents^5^ and to compare the two tests in our setting.

## Methods

### Population and study site

Seventy-three children and adolescents who had been booked for upper gastrointestinal endoscopy were recruited into the study after obtaining consent from their caregivers. This study was conducted in the Paediatric Endoscopy Unit, located in the paediatric endoscopy unit, located in the paediatric theatre of the Korle Bu Teaching Hospital in Accra, Ghana, from March to November 2018. This unit provides endoscopy service for patients within the hospital, as well as those referred from other facilities in the country or sub-region.

### Exclusion criteria

Patients who had taken proton pump inhibitors and/or antibiotics in the preceding two and four weeks were excluded from the study.

### Endoscopy and Histology Procedures

Two invasive tests were used to diagnose the presence of *H. pylori* infection. The procedure was done under conscious sedation with intravenous midazolam and ketamine. Six biopsies were taken from the antrum and body of the stomach for each patient. Two of these biopsies were impregnated on the RUT kit (Pronto Dry®), which was read by the endoscopist at 5 and 30 minutes using the colour scale provided by the manufacturer. In samples that were negative in the first 30 minutes, they were read again in one hour to confirm the negativity of the result. The rest of the biopsies were placed in 10% buffered formalin solution in a specimen container appropriately labelled and transported to the histopathology laboratory. The histopathologist was blinded to the result of the RUT. The biopsies were processed routinely into formalin fixed paraffin embedded blocks; slides were prepared from thin sections (3um) and stained with Hematoxylin and Eosin, Giemsa and special stains. The stained slides were examined microscopically for the presence of *H. pylori-associated* gastritis.

### Patients' information and risk factors

Each patient's gender, age, indication for endoscopy, type of housing they were living in and the presence of pets in the house were obtained.

### Ethical approval

Ethical approval (KBTH-STC 00054/2017) was obtained from the Institutional Review Board of the Korle Bu Teaching Hospital. First, caregivers' consent was obtained, and verbal assent was obtained from children 7 years of age and above.

### Statistical Analysis

Data were captured on a Microsoft Excel spreadsheet and exported to Stata version 13.0 for analysis. Frequencies and percentages were determined for categorical variables, whilst numeric variables were summarized using means and their corresponding standard deviations. To determine associations among categorical variables, cross-tabulation was done Pearson's chi-square values were computed and their corresponding p-values determined. Simple logistic regression models were fitted to the data using each of the dichotomised patient characteristics as a predictor variable and one of the diagnostic tests as the outcome variable. Crude odds ratios (e β) were computed in each of the bivariate models as a measure of the strength of association between test results and the selected predictor. Using the simple logistic regression model with rapid urease test result as an outcome variable and each of the patients' characteristics in the first column of Table 3 as a predictor variable, we obtained the first set of crude odds ratios with corresponding p-values shown in Table 3.

**Table 3:** Association of Rapid Urease Test (RUT) and the presence of *H. pylori* associated gastritis

	Histology presence of *H. pylori* n(%)		p-value*	Gastritis n(%)		p-value*
**RUT**	Yes	No		Chronic gastritis	No gastritis	
**Positive**	36(85.7)	6(14.3)	<0.001	42 (100.0)	0(0)	<0.001
**Negative**	3(9.7)	28(90.3)		11 (35.5)	20 (64.5)	

* p-value for chi-square test

Repeating the analysis with the same model but this time using the histologic presence of *H. pylori* in gastric biopsy as the outcome variable, we obtained the second set of crude odds ratios and corresponding p-values shown in Table 3. Adjusted odds ratios (AOR) were computed in a multiple logistic regression model using each of the diagnostic tests as an outcome variable at a time and the dichotomised patients' characteristics as covariates in the model (see Table 4). Cohen's Kappa statistic was also computed to determine agreement between the two diagnostic tests. Statistical significance was determined at p<0.05.

**Table 4:** Bivariate logistic regression analysis using rapid urease test and histology as outcome variables

Rapid Urease Test					*H. pylori* associated gastritis			
**Variable**	**Positive**	**negative**	**Crude Odds Ratio**	**p-value**	**Positive**	**Negative**	**Crude Odds Ratio**	**p-value**
**Age(yrs)**								
**<5**	**5**	**4**	**0.91**	**0.898**	**4**	**5**	**0.66**	**0.564**
**≥5 (ref)**	**37**	**27**	**1**		**35**	**29**	**1**	
**Sex**								
**Male**	**24**	**20**	**0.73**	**0.525**	**23**	**21**	**0.89**	**0.808**
**Female (ref)**	**18**	**11**	**1**		**16**	**13**	**1**	
**Household size**								
**≥6**	**21**	**9**	**2.44**	**0.072**	**22**	**8**	**4.21**	**0.006** *
**<6 (ref)**	**21**	**22**	**1**		**17**	**26**	**1**	
**Type of housing compound**	**14**	**12**	**0.79**	**0.635**	**15**	**24**	**1.31**	**0.587**
**Self-cont'd (ref)**	**28**	**19**	**1**		**11**	**23**	**1**	
**Presence of pets**								
**Yes**	**15**	**8**	**1.60**	**0.368**	**17**	**6**	**3.61**	**0.021** *
**No (ref)**	**27**	**23**	**1**		**22**	**28**	**1**	

ref=reference category

* Sigmficant at p≤0.05

## Results

Seventy-three children were seen during the period. Their ages ranged from 2 to 16 years, with a median age of 9 years. As shown in Table 1, they were predominantly males (60 %), lived in self-contained (nuclear family only) houses (64 %), and came from households of size less than 6 members (59 %). Most (68 %) did not have pets in their houses.

**Table 1:** Demographic and household characteristics of the participants

Demographic characteristics	Number(%), n= 73
**Age (years)**	
**0-4**	9(12.3)
**5-9**	33(45.2)
**10-14**	28(38.4)
**15-19**	3(4.1)
**Mean**	8.8
**Standard Deviation**	3.3
**Sex**	
**Males**	44(60.3)
**Females**	29(39.7)
**Type of housing**	
**Compound**	26(35.6)
**Self-contained**	47(64.4)
**Household size**	
**Less than 6**	43(68.9)
**6 or more**	30(41.1)
**Type of Pet**	
**Cats**	12(16.4)
**Dogs**	6(8.2)
**Cats and Dogs**	5(6.9)
**No pets**	50(68.5)

### Indications for endoscopy

The commonest indication for endoscopy was abdominal pain in 41 (56.1%) patients; this was followed by gastrointestinal bleeding in 12 (16.4%), vomiting in 8 (11.0%), heart burns in 8(11.0%) and failure to thrive in 4 (5.5%) patients.

### Endoscopic appearance of the stomach

Forty patients (54.8%) had normal-appearing gastric mucosa. Nodularity was the commonest anomaly seen in 19 (26.0%) children at endoscopy. Twelve (16.4%) were localised in the antrum, while 7(9.6%) were diffuse nodularity in the stomach. Other lesions observed included erythema with haemorrhages in 6 (8.2%), erosions in 5 (6.8%) and ulcers in 3 (4.1%) patients.

In order to show agreement, if any, between the two diagnostic tests, RUT results were cross-tabulated with histopathology results, as shown in Table 2. Both tests were positive at the same time in 36 (49.3%) out of the 73 children seen. The positivity rates for RUT and histology examination of gastric biopsies were 57.5% and 53.4%, respectively. The two tests were discordant in 9(12.3%) out of 73 children. When Cohen's Kappa (κ) was run to determine if there was agreement between the two tests, the analysis showed substantial agreement of 87.7 %, κ =0.751 (p<0.0001).

**Table 2:** Rapid urease test results against histologic examination for *H. pylori*

	Histology presence of *H. pylori*		
**Rapid Urease** **Test**	**Yes**	**No**	**Total**
**Positive**	36	6	42
**Negative**	3	28	31
**Total**	39	34	73

Using histology examination as a confirmatory test, the sensitivity and specificity of RUT was estimated as being 92.3% and 82.4% respectively.

There was a significant association (p-value for chi-square test <0.05) between positive rapid urease test results and the presence of chronic gastritis. Chronic gastritis here implies both chronic active and chronic inactive gastritis. All those who tested positive with the RUT had chronic gastritis (17 chronic active and 25 chronic inactive gastritis). Amongst those who tested negative with RUT, 10 patients had chronic inactive gastritis whilst just one patient had chronic active gastritis.

There was no significant association between RUT results and any of the predictor variables of socioeconomic status (i.e. age, sex, household size, type of housing, presence of pets, and whether or not child had been on medications). However, when the histology presence of H. pylori was made the outcome variable in the model, a significant association was found between household size as well as the presence of pets and the histologic presence of *H. pylori* in gastric mucosa. The crude odds of having *H. pylori* present in gastric mucosal biopsy was more than four times higher among children from larger households (with at least six members) compared with those from households with smaller sizes (Crude OR:4.21; p<0.006). Children living with pets were also 3.6 times as likely to present with H. pylori-associated gastritis. Likewise, in a multiple logistic regression model with histology presence of *H. pylori* in the stomach as the outcome variable, large household size (6 or more) and the presence of pets at home were found to be significant predictors of a positive histology result.

## Discussion

In this study, we set out to detect the presence of *H. pylori* infection in children reporting for upper gastrointestinal endoscopy using two diagnostic methods (rapid urease test and histology) and to explore the extent to which the two commonly used diagnostic tests agreed with each other. We also explored the relationship between each of the two diagnostic test results and indicators of socioeconomic status.

The findings showed *H. pylori* infection in 53.4% (by histopathology) and 57.5% (by rapid urease test) of the children. The rate of infection is comparatively higher than previous studies in children in Ghana 10 and Ethiopia^11^, with rates of infection at 14.2% and 34.6%, respectively. These two African studies were done in asymptomatic children, used faecal stool antigen test, which has a lower sensitivity and specificity compared to RUT.

The relatively lower positivity rate for histopathology compared with rapid urease test is consistent with previous studies that have sought to compare the performance of the two diagnostic tests.^12^ Other urease-producing organisms (Proteus mirabilis, Citrobacter freundii, Klebsiella pneumonia, Enterobacter cloacae and Staphylococcus aureus) present in the gastric biopsy or prolonged contact of the biopsy with the RUT medium longer than 24 hours, could cause a false positive RUT and hence a lower histopathology positivity rate.^8^ Within the constraints of a small sample size, this finding seems to support the need for a combination of the two tests as a gold standard^5^ whenever possible. However, the substantial agreement observed between the two tests seems to give some confidence to the clinician working under unfavourable conditions in a resource-limited environment to use just the rapid urease test result and clinical judgments to inform management decisions. This approach should, however, be adopted cautiously in view of the seemingly high “false positive” rate (14.3%) of the rapid urease test in this study. This could be due to the small sample used in this study. The need for a larger study is thus highly indicated.

Worthy of note is also the observation that a large household size of at least 6 members (AOR: 4.03; p<0.013) and/or the presence of pets at home (AOR:3.23; p<0.044) could significantly predict the histologic presence of H. the in gastric mucosa. The exact routes of *H. pylori* infection are still not known. There is some supportive biologic and epidemiologic evidence that transmission may occur through multiple pathways, both from person to person and through external sources, with dominant routes perhaps varying between different populations. 13 Also, a number of helicobacter species, including H. pylori, have been detected in the gastric mucosa of pet animals, including dogs, with prevalence ranging from 67–86% in clinically healthy dogs and 61–100% in animals presenting with chronic vomiting.^14^ Person-to-person transmission is most commonly implicated with faecaloral, oral-oral, or gastric-oral pathways.^15^ Early colonization in children living under poor socio-economic conditions has been demonstrated, and several studies have shown a high prevalence of *Helicobacter pylori* among people in low-income countries. Low-income societies are often associated with large household sizes and poor living conditions that might favour person-person transmission.

Recognising the constraints of small sample sizes on multiple logistic regression models, we recommend a larger study that will allow one to control for more indicators of socioeconomic factors such as income levels, occupation and educational level of parents.

This study is largely limited by the fact that it was performed on symptomatic patients in a hospital setting. Hence, this cannot be a true reflection of the prevalence of *H. pylori* within the community as this is commonly an asymptomatic infection in children. The small sample size, the absence of a gold standard as a confirmatory test, and resource constraints are issues that we hope to address in a larger study, given adequate funding.

## Conclusion

About half of the children presenting for Upper GIT Endoscopy in Korle Bu Teaching Hospital for various indications had *H. pylori* infection. Children from large households and those with pets at home have increased odds of having *H. pylori* infection of the gastric mucosa. Also rapid urease test agreed substantially with histology in the diagnosis of *H. pylori* infection. In resource-constrained settings where there may not be a pathologist, the RUT alone is recommended as a confirmatory test for the presence of *H. pylori*. We recommend further studies to throw more light on these preliminary findings.
